# Integrating molecular and conventional diagnostics in native vertebral osteomyelitis: a narrative review

**DOI:** 10.5194/jbji-11-373-2026

**Published:** 2026-06-29

**Authors:** Farzad Pourghazi, Omar Mahmoud, Francesco Petri, Said El Zein, Gina A. Suh, Takahiro Matsuo, Andrea Gori, Audrey N. Schuetz, Elie F. Berbari

**Affiliations:** 1 Division of Public Health, Infectious Diseases and Occupational Medicine, Department of Medicine, Mayo Clinic College of Medicine and Science, Mayo Clinic, Rochester, Minnesota, 55905, USA; 2 Department of Infectious Diseases, ASST Fatebenefratelli Sacco University Hospital, Regional Center for Infectious Diseases (CEREMI), Lombardy Region, Milan, Italy; 3 Department of Infectious Diseases, Infection Control, and Employee Health, The University of Texas MD Anderson Cancer Center, Houston, Texas, 77030, USA; 4 Department of Biomedical and Clinical Sciences, University of Milan, Milan, 20157, Italy; 5 Centre for Multidisciplinary Research in Health Science (MACH), University of Milan, Milan, 20157, Italy; 6 Division of Clinical Microbiology, Department of Laboratory Medicine and Pathology, Mayo Clinic, Rochester, Minnesota, 55905, USA

## Abstract

Native vertebral osteomyelitis (NVO) remains a diagnostic challenge due to its non-specific presentation and the limited sensitivity of culture-based methods. Blood cultures and image-guided biopsies are considered the diagnostic standards, but their yield is often low, especially in patients who have received prior antibiotics or are infected with fastidious microorganisms. Recent molecular techniques, including polymerase chain reaction (PCR) and next-generation sequencing (NGS), have improved pathogen detection and enabled more targeted antimicrobial therapy.

In this narrative review, we summarize the diagnostic methods used in NVO, including conventional microbiological and molecular approaches, and present their strengths and limitations based on previous studies. We particularly focus on situations where molecular diagnostic techniques, such as 16S rRNA PCR, NGS, and microbial cell-free DNA testing, outperform traditional culture-based methods in sensitivity, while their specificity may be comparatively lower. Combining these molecular tools with standard diagnostic procedures may improve pathogen detection, guide targeted treatment, and enhance diagnostic accuracy in culture-negative or antibiotic-pretreated cases. Understanding the advantages and limitations of each diagnostic method can help clinicians to choose the most appropriate testing strategy, reduce diagnostic delays, and improve patient management in suspected NVO.

## 1 Introduction

Native vertebral osteomyelitis (NVO) accounts for approximately 3 %–5 % of all osteomyelitis cases (Kremers et al., 2015) and can result in severe complications, including irreversible spinal deformity, spinal cord injury, neurological deficit, or even death (Nagashima et al., 2018). In a recent nationwide study, Channer et al. (2025) analyzed data from 1 million adults and identified 1469 cases of NVO between 2010 and 2020, giving an annual incidence of 14.69 per 100 000 individuals. That study found stimulant use (such as methamphetamine use) to be the strongest risk factor, followed by diabetes, obesity, chronic liver disease, tobacco use, alcohol use, and cannabis use (Channer et al., 2025). These recent findings show a significant increase in the incidence of NVO in recent years, which may also reflect improvements in diagnostic accuracy and case ascertainment, as well as demographic changes such as population aging, increased multi-morbidity, and the rising number of spinal diseases and surgical interventions (Nickerson and Sinha, 2016). Together, both studies suggest that medical comorbidities, lifestyle-related factors, and enhanced diagnostic methods contribute to the increasing occurrence of NVO in the United States.

The main clinical manifestation of NVO is persistent localized back pain that does not respond to conventional therapy (Berbari et al., 2015). However, non-specific clinical signs and laboratory markers often complicate the diagnosis of NVO, leading to significant delays in treatment (Nagashima et al., 2018). The Infectious Diseases Society of America (IDSA) advises a combination of serologic, radiologic, and microbiologic tests for evaluating patients suspected of having NVO (Berbari et al., 2015). Timely and accurate diagnosis, including identification of the causative microorganism and its susceptibility patterns, is critical for the optimal management of NVO (Maamari et al., 2022). Nonetheless, diagnosing NVO remains challenging, as culture-based methods identify the causative organism in only about 30 %–70 % of cases, depending on the specimen type and prior antibiotic use (Kim et al., 2015). This issue is exacerbated in patients who have already received antibiotics or when dealing with fastidious microorganisms, often resulting in culture-negative NVO, which results in an inability to provide input on pathogen-directed therapy (Alavi et al., 2024). This limitation carries risks such as unnecessary antimicrobial use or the administration of inappropriate antibiotics.

Molecular techniques may offer detailed microbial profiling and can reduce the time required to identify the infecting organism when used early in the diagnostic workflow and when available on site. However, their turnaround time varies substantially by platform and setting. For example, commercial multiplex PCR panels can provide rapid results, although they are not specifically licensed for vertebral biopsy specimens or non-synovial abscess fluids, whereas more complex shotgun metagenomic next-generation sequencing (NGS) approaches may require additional time for analytical processing and interpretation. Therefore, the optimal role of each molecular test should be aligned with its expected turnaround time, local availability, and clinical context, and further studies are needed to define their most appropriate use.

Recent meta-analysis found moderate to high diagnostic performance of molecular methods on direct patient specimens for the diagnosis of NVO (Mahmoud et al., 2025). This emphasizes the use of these methods in combination with conventional methods for NVO diagnosis. Due to the increasing incidence of NVO, the challenges associated with its diagnosis, and the risk of delayed treatment, this narrative review provides an overview of the available diagnostic approaches, with a focus on microbiological and recent molecular diagnostic techniques such as 16S rDNA PCR and NGS. The aim of this review is to summarize these diagnostic modalities, focus on the role of molecular methods within current diagnostic frameworks, and discuss how they may enhance pathogen detection and improve the clinical management of NVO.

## 2 The IDSA 2015 guidelines for the microbiological diagnosis of NVO

Blood cultures and image-guided biopsies remain central to the microbiological diagnosis of NVO. Blood culture positivity rates range from approximately 30 % to 73 % across studies (Colmenero et al., 2010; Lacasse et al., 2023; Matsuo et al., 2026), highlighting their important but variable diagnostic yield. The 2015 IDSA guidelines indicate that, in patients with compatible clinical and imaging findings, positive blood cultures for selected pathogens (e.g., *Staphylococcus aureus*, *Staphylococcus lugdunensis*, or *Brucella* spp.) may be sufficient to establish the diagnosis without the need for image-guided biopsy (Berbari et al., 2015). In clinical practice, however, other bloodstream isolates, including β -hemolytic streptococci and Gram-negative bacilli, may also support the diagnosis in appropriate contexts and help to avoid invasive procedures in selected patients.

**Figure 1:**
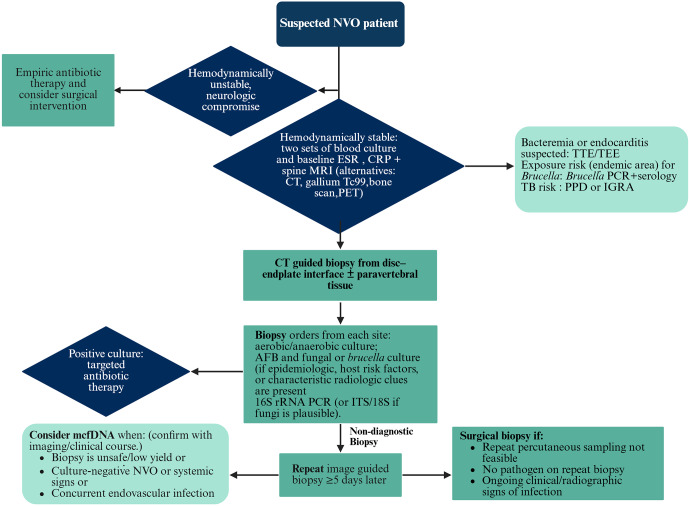
Suggested diagnostic approach for suspected NVO, adapted from the 2015 IDSA guidelines and incorporating recent advances in molecular diagnostics. Created in BioRender. F. Pourghazi (2026), https://BioRender.com/prnkrpm (last access: 26 June 2026). Abbreviations: AFB: acid-fast bacilli; CRP: C-reactive protein; CT: computed tomography; ESR: erythrocyte sedimentation rate; IGRA: interferon-gamma release assay; ITS: internal transcribed spacer; mcfDNA: microbial cell-free DNA; MRI: magnetic resonance imaging; NVO: native vertebral osteomyelitis; PCR: polymerase chain reaction; PET: positron emission tomography; PPD: purified protein derivative; rRNA: ribosomal RNA; TEE: transesophageal echocardiography; TTE: transthoracic echocardiography.

Consistent with guideline recommendations (Berbari et al., 2015; Lacasse et al., 2023), at least two sets of bacterial blood cultures, each including paired aerobic and anaerobic bottles, should be obtained in all patients with suspected NVO, before the initiation of antimicrobial therapy when clinically feasible. In instances where blood cultures do not detect any pathogens, an image-guided biopsy is advised to determine the causative agent (Berbari et al., 2015). Specimens should be submitted for microbiology and histopathology examination. Additionally, fungal and mycobacterial etiologies should be considered in patients with culture-negative NVO, immuno-suppressed status, or risk factors such as living in endemic areas (Berbari et al., 2015). For *Brucella* spp., the diagnostic workup should include paired serologic testing, using agglutination assays and enzyme-linked immunosorbent assays (ELISA), together with targeted PCR when available, and blood cultures containing beads or resins which neutralize antibiotics, as well as prolonged incubation times of up to 14 d, which may improve organism recovery (Di Bonaventura et al., 2021). In cases of suspected tuberculosis, including patients with compatible clinical features (subacute or chronic course, fever, weight loss) or supportive radiologic findings (such as thoracic involvement), an interferon- γ release assay or purified protein derivative test may be performed and can aid in the diagnostic evaluation, recognizing their limitations (Berbari et al., 2015). However, the diagnosis of tuberculous NVO is mainly established by microscopy and culture of infected tissue. Figure 1 illustrates the IDSA diagnostic algorithm for NVO.

Similar recommendations are shared in other European society documents, such as the joint European Association of Nuclear Medicine and European Society of Neuroradiology (EANM/ESNR) and European Society of Clinical Microbiology and Infectious Diseases (ESCMID) endorsement consensus (Lazzeri et al., 2019), and the French 2022 Société de Pathologie Infectieuse de Langue Française (SPLIF) guidelines (Lazzeri et al., 2019; Lacasse et al., 2023).

In the following sections, we discuss the different diagnostic modalities for NVO, highlighting their strengths, limitations, clinical implications, and recent advancements in each.

### 2.1 Biopsy

The two most widely recognized methods for biopsy in the context of NVO are image-guided percutaneous biopsy and open biopsy (McNamara et al., 2017). The yield of percutaneous biopsy has been reported to range between 48 % and 52 % in recent meta-analyses (McNamara et al., 2017; Pupaibool et al., 2015), whereas open biopsy has a higher yield of 76 % (McNamara et al., 2017; Pupaibool et al., 2015). However, due to the complex anatomy of the spine, image-guided biopsy is preferred for percutaneous sampling of NVO, since it is safer and less invasive (Berbari et al., 2015). However, this method can be limited by its low sensitivity (Sehn and Gilula, 2012). Furthermore, sampling error is a recognized limitation, as infected tissue may be difficult to access or focal in distribution (Berbari et al., 2015). Although the sensitivity is limited, repeat blood cultures after CT-guided biopsy may be considered as a simple and low-cost supplemental approach to improve microbiological yield in selected cases (Lacasse et al., 2023).

#### 2.1.1 Specimen number, type, and location

When feasible, a biopsy can be directed toward structures such as bone, disk, and adjacent infected spinal sites like facet joints or paraspinal soft tissues. However, the impact of targeting these different tissue structures during percutaneous biopsy on microbiological yield remains uncertain. In a retrospective study by Chang et al. (2015) involving 111 biopsies, there was no significant difference in microbiological yield when sampling paravertebral soft tissue, disk tissue, or a combination of disk and vertebral endplate. Conversely, Kim et al. (2015) demonstrated a higher microbiological yield when specimens were taken from disks and abscesses compared to bone tissue, likely due to the rigidity of bone and the difficulty in adequately homogenizing bone specimens for plating and culture. A study by Beroukhim et al. (2019) reported that lower bone density was associated with higher rates of positive cultures, suggesting that the physical characteristics of bone may affect culture yield. Similarly, Maamari et al. (2022) found that image-guided biopsies had three times higher odds of detecting a microorganism when fluid was successfully aspirated, compared to cases of dry aspiration, in instances where imaging suggested a fluid collection.

Two other factors that may influence the yield of percutaneous biopsies are the number of biopsies taken and the choice of needle. Although studies have provided conflicting results, it is generally recommended to use larger inner-bore needles (at least 14-gauge) whenever possible to maximize yield, provided safe access to abnormal tissue is feasible (Husseini and Huang, 2023). However, the Maamari et al. study reported that needle gauge did not significantly influence diagnostic yield (Maamari et al., 2022). Despite some evidence from studies conducted by Maamari et al. (2022) and Chang et al. (2015) indicating that the number of specimens taken during the biopsy is not associated with a higher yield, a study by Husseini and Huang (2023) recommended that operators should consider maximizing the number of biopsy specimens that can be safely acquired following a single placement of the introducer needle. However, repositioning the needle may be associated with a higher risk of injury, with limited evidence of additional benefit (Husseini and Huang, 2023).

Beyond tissue selection, several procedural and clinical factors influence the microbiological yield of biopsy in NVO, which are discussed in the following sections. In addition to pre-analytic factors, the optimization of culture methodology is critical to maximizing microbiological yield in NVO. Key considerations include adequate incubation duration, with extended incubation warranted for fastidious or slow-growing organisms (e.g., *Cutibacterium acnes* and certain anaerobes), the role of post-biopsy blood cultures, as well as the selective use of enrichment broths to enhance recovery in low-inoculum infections. Inoculation onto both aerobic and anaerobic media and, when clinically indicated, specialized media for mycobacteria or fungi should be guided by the clinical context. Close collaboration with the microbiology laboratory is essential to tailor processing protocols, including incubation conditions and duration, to the suspected pathogens.

#### 2.1.2 Prior antimicrobial exposure

The IDSA recommendations suggest withholding antimicrobial therapy before vertebral biopsy for 1–2 weeks, except in cases where the patient presents with neurological deficit or hemodynamic instability. Immediate surgical treatment with antimicrobial therapy is recommended in these two cases (Berbari et al., 2015). However, data on the impact of prior antimicrobial treatment on the yield of culture results by biopsy remain conflicting. A previous meta-analysis in 2017, which assessed the yield of open and percutaneous biopsy, showed a decrease in image-guided biopsy culture yield from 43 % to 32 % when patients had received antibiotics before biopsy compared to those with no antimicrobial therapy before sampling (McNamara et al., 2017). However, these results did not reach statistical significance (McNamara et al., 2017). In a recent large cohort study, Winkler et al. found no statistically significant association between prior antibiotic exposure and positive microbiological culture yield, although a trend toward reduced yield was observed (Winkler et al., 2024). Two more recent studies have also shown similar results, with no effect of prior antibiotic exposure on biopsy yield by culture in patients with suspected NVO (Hoang et al., 2019; Schiro et al., 2020). Conversely, in a retrospective study by Maamari et al. (2022) where image-guided biopsy yielded a positive culture result in 52.6 % of patients, antimicrobial exposure before the procedure was significantly associated with three-fold decreased odds of a positive yield compared to patients who did not receive antibiotic treatment before biopsy. A gradual increase in microbiological yield was observed with longer antibiotic-free intervals, with recovery rates improving notably after approximately 4 antibiotic-free days. Overall, the effect of prior antibiotic exposure on the diagnostic yield of biopsy procedures remains uncertain and requires further investigation.

#### 2.1.3 Repeat biopsy

Current IDSA guidelines advocate for a repeat biopsy in suspected NVO for cases in which the initial image-guided biopsy yielded negative or non-diagnostic results such as skin contaminants (Berbari et al., 2015) (Fig. 1). If necessary, a repeat biopsy should be collected at least 5 d after the initial biopsy since most organisms are recovered by culture within 3 d (Yeh et al., 2020). However, the yield of a repeat biopsy may be diminished compared to the initial one, particularly in patients already undergoing antimicrobial therapy (Czuczman et al., 2018). In addition, repeat biopsy may introduce delays in the initiation of antimicrobial therapy and can be associated with patient discomfort, particularly in those with significant pain, which should be considered when weighing its clinical utility. Alternatively, if the initial image-guided biopsy yields negative results, an open biopsy could be considered as the subsequent step (Berbari et al., 2015). Weihe et al. (2022) reported that repeat image-guided percutaneous biopsy increased the overall diagnostic yield by approximately 13 %, supporting its use in cases with an initial non-diagnostic result. Therefore, the decision to pursue repeat biopsy should be individualized, balancing potential diagnostic benefit against procedural burden and potential delays in treatment.

#### 2.1.4 Biopsy complications

A recent systematic review and meta-analysis evaluated the safety profile of image-guided vertebral biopsy, including 39 studies with a total of 3917 patients who underwent 4181 procedures (Michalopoulos et al., 2021). Across these studies, 67 complications were reported, corresponding to an overall complication rate of 1.0 % (95 % CI 0.4 %–1.9 %). Major complications accounted for 8 cases (0.2 %) and included spinal cord compression ( n=3 ), tuberculous fistula formation along the biopsy tract ( n=3 ), and hematoma requiring surgical evacuation or transfusion ( n=2 ). The remaining 59 cases were minor complications, most commonly transient pain, small hematoma, or temporary neurological symptoms (Michalopoulos et al., 2021). Importantly, no cases of tumor seeding were observed. The authors also identified a significant decline in complication rates across publication years of the included studies (Spearman R=-0.39 , p=0.034 ), showing progressive improvement in procedural safety with increased operator experience and modern imaging techniques (Michalopoulos et al., 2021).

### 2.2 Role of inflammatory biomarkers in the diagnosis of NVO

Erythrocyte sedimentation rate (ESR) and C-reactive protein (CRP) are highly sensitive for NVO and should be obtained in all suspected cases (Berbari et al., 2015; Zimmerli, 2010). However, these biomarkers still lack specificity and may be elevated for reasons other than infection (Berbari et al., 2015). CRP > 10 mg L^−1^ has high sensitivity for NVO, whereas CRP > 4 × the upper limit of normal are associated with a positive biopsy culture, with a sensitivity of 91 % and specificity of 74 % (Davis et al., 2020; Lee et al., 2020).

Beyond diagnosis, inflammatory markers may also have prognostic value. The initial ESR value and the variability in ESR during the first 4 weeks have been reported to be useful markers for predicting treatment duration and recurrence (Chiang et al., 2019). In contrast, white blood cell (WBC) count has limited utility for follow-up, as it is elevated in only 64 % of cases at diagnosis and does not correlate well with clinical response (Jensen et al., 1998).

Procalcitonin (PCT) offers high specificity (0.90) but limited sensitivity (0.67), and levels > 0.11 ng mL^−1^ have been associated with infection persistence (OR 3.78), suggesting potential prognostic utility (Shen et al., 2013; Santagada et al., 2022). In addition, PCT levels are significantly higher in pyogenic than tuberculous spondylodiscitis, contributing to combined prediction models (AUC 0.93) for differentiating these entities (Yoon et al., 2015).

### 2.3 Molecular techniques

Molecular diagnostic methods, such as PCR and NGS, primarily contribute to the identification of microbial etiology rather than establishing the diagnosis of NVO, which relies on clinical, laboratory, and imaging findings. These techniques can supplement culture-based approaches, overcome some limitations of traditional methods, and improve accuracy in identifying causative microorganisms in NVO.

#### 2.3.1 PCR

**Figure 2:**
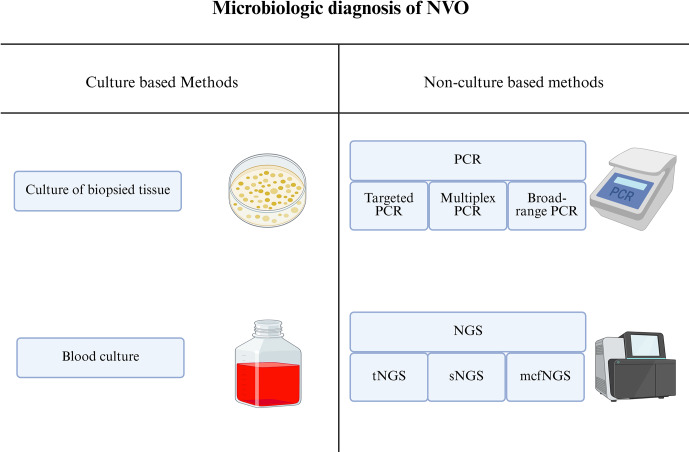
Different microbiologic techniques used for pathogen identification in NVO. Abbreviations: mcfNGS: microbial cell-free DNA next-generation sequencing; sNGS: shotgun next-generation sequencing; tNGS: targeted next-generation sequencing. Created in BioRender. F. Pourghazi (2026), https://BioRender.com/e5hb4o6 (last access: 26 June 2026).

PCR has been utilized in multiple domains of infection diagnostics. PCR may be run directly on a patient specimen, or it may be used as an identification method for an isolate that has been recovered by culture. The main three types of PCR used in the diagnosis of osteoarticular infections are (1) PCR using primers and probes to detect a particular microorganism (also called targeted PCR), (2) using a multiplex panel used for simultaneous detection of several microorganisms by adding primers and probes of interest (also called multiplex PCR), or (3) broad-range PCR (also called universal) followed by either Sanger sequencing or NGS to identify the amplified product (Higgins et al., 2022) (Fig. 2).

Multiplex real-time PCR is a targeted approach using organism-specific primers. In one study, a multiplex assay differentiating tuberculous from brucellar vertebral osteomyelitis achieved 93.3 % sensitivity and 90 % specificity, outperforming conventional cultures (Colmenero et al., 2010).

**Figure 3:**
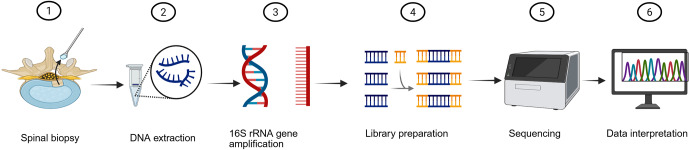
Workflow of 16S/bacterial broad-range PCR. Created in BioRender. F. Pourghazi (2026), https://BioRender.com/0sk2y0b (last access: 23 June 2026).

Broad-range PCRs, using primers targeting the 16S rRNA gene and/or 23S rRNA gene, are particularly used to detect bacteria (Fenollar and Raoult, 2004). However, in fungal infections, species identification is based on the analysis of the 18S, 5.8S, and 28S gene sequences. The internal transcribed spacer (ITS) region also serves as a universal fungal target for broad-range PCR and allows for the differentiation between different fungal species (Kullar et al., 2023; Reller et al., 2007). However, fungal broad-range PCR is not widely available. The 16S bacterial broad-range PCR process consists of three stages: amplification and visualization of the amplified products, sequencing of the amplified product upon a positive PCR result, and subsequent analysis and comparison of the sequence with a database containing all known bacterial sequences to ensure accurate identification (Fenollar et al., 2008) (Fig. 3). Broad-range PCR is a molecular diagnostic technique that uses universal primers targeting conserved regions of bacterial or fungal DNA, allowing the detection of a wide range of pathogens without prior assumptions. The amplified DNA is subsequently sequenced and compared with reference databases to accurately identify the species involved.

This procedure should be conducted on fresh specimens or paraffin-embedded tissue blocks obtained from fluids or bone biopsies via needle aspiration or surgical biopsy. However, in some settings where PCR is performed only after negative culture results, specimens may be stored (e.g., frozen) prior to testing. 16S PCR can also be performed on clinical isolates recovered in culture.

A recent meta-analysis by Mahmoud et al. (2025) reported a sensitivity of 78 % and a specificity of 94 % for broad-range 16S rRNA PCR in diagnosing NVO when evaluated against a composite reference standard that combined microbiological, clinical, laboratory, and imaging criteria to define true infection (Mahmoud et al., 2025). In earlier studies, 16S PCR demonstrated high sensitivity when compared to culture or histopathology on tissue biopsy (Choe et al., 2014), exceeding the performance typically observed with culture alone (McNamara et al., 2017; Chew and Kline, 2001; Pupaibool et al., 2015). This higher diagnostic performance of 16S rRNA PCR, as shown in the recent meta-analysis, is mainly due to its ability to detect bacterial DNA without depending on organism viability. It can identify pathogens in specimens even after antibiotic treatment has begun or when infections are caused by fastidious or slow-growing bacteria. These features explain its high sensitivity and support its use as a diagnostic tool for NVO complementary to culture. Recent advances in this method have improved its accuracy and speed, enabling the reliable detection of fastidious, slow-growing, or unexpected organisms directly from clinical specimens (Asthana et al., 2024). Additionally, in cases of culture-negative NVO, where standard bacterial cultures fail to identify pathogens, often due to prior antibiotic exposure, broad-range 16S rRNA PCR has shown considerable diagnostic value. Recent evidence using broad-range 16S rRNA PCR with sequencing reported a diagnostic sensitivity of 88.5 % and specificity of 83.5 % for the detection of bacterial pathogens in normally sterile orthopedic samples with a higher detection rate than conventional culture (34.6 % versus 25 %). Importantly, PCR identified 13 culture-negative infections, largely attributable to the presence of fastidious organisms and prior antibiotic exposure. These findings highlight the important role of 16S rRNA PCR as a supplemental tool in improving the microbiological diagnosis of culture-negative osteoarticular infections, including cases of spondylodiscitis and suspected NVO (Grif et al., 2012). For example, Fuursted et al. reported that 16S rRNA PCR could help to detect a microorganism in an additional 44 % of culture-negative cases, underscoring its effectiveness in detecting spinal infections caused by fastidious bacteria such as *Kingella kingae* and *Clostridium histolyticum* (Fuursted et al., 2008).

However, PCR is not without its challenges – mainly the risk of false positives due to the detection of DNA fragments instead of live pathogens and because of possible contamination. Contamination often occurs during specimen collection, for example, when bacteria from the skin enter the specimen during biopsy (Passerini et al., 2023). This highlights the importance of maintaining clean and sterile sampling conditions (Mahmoud et al., 2025). Other sources of contamination include laboratory handling errors, contact with unclean equipment or surfaces, and cross-contamination between specimens, especially when testing is performed in an open system. Performing PCR in a closed system, where amplification and identification occur in one container, can help to reduce this risk (Fenollar et al., 2008; Fenollar and Raoult, 2004). Recent meta-analysis reported that the specificity of 16S rRNA PCR is about 94 %, with an estimated false-positive rate of around 6 %, mostly caused by environmental or reagent contamination or by amplifying non-viable bacterial DNA. Careful contamination control during specimens collection and laboratory processing is therefore essential (Mahmoud et al., 2025). Preventive measures include wearing gloves and protective coats, cleaning the work area after each run, and using separate rooms for specimen preparation and amplification (Fenollar et al., 2008).

A major limitation of broad-range PCR followed by Sanger sequencing is that it cannot provide information on antimicrobial susceptibility. Also, sequence analysis can sometimes be misleading, especially in polymicrobial infections, because Sanger sequencing usually detects only one dominant organism, while NGS can detect several. Furthermore, sequencing may be less effective in polymicrobial specimens because overlapping sequence reads can make interpretation difficult (Mahmoud et al., 2025).

#### 2.3.2 NGS

**Figure 4:**
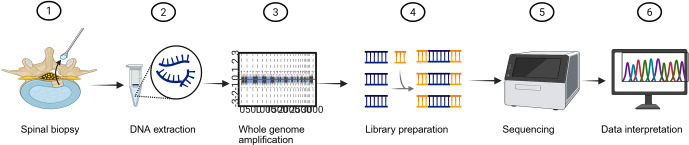
Workflow of next-generation sequencing (NGS). Created in BioRender. F. Pourghazi (2026), https://BioRender.com/wlk1m1w (last access: 26 June 2026).

NGS is a high-throughput technology that can sequence millions of DNA fragments simultaneously, allowing comprehensive genetic analysis of microorganisms in clinical specimens. NGS methods are generally classified as targeted NGS (tNGS) or shotgun NGS, which is referred to as metagenomic NGS (mNGS) when applied to mixed clinical specimens containing both host and microbial nucleic acids. tNGS allows the sequencing of specific genetic markers, such as the bacterial 16S rRNA gene or certain conserved regions used for fungal identification, such as the internal transcribed spacer (ITS). In contrast, shotgun NGS (or mNGS) sequences all nucleic acids in a specimen without prior selection, allowing the identification of any microbial DNA or RNA present, including unexpected or novel pathogens (Hu et al., 2021; Flurin et al., 2022). The general workflow of NGS involves several sequential steps, including specimen collection (for example, spinal biopsy), DNA extraction, whole-genome amplification, library preparation, sequencing, and bioinformatic data interpretation (Fig. 4). These methods have expanded the ability to detect pathogens directly from clinical material (Gu et al., 2019).

This technique has significantly improved microbial diagnostic efficiency in patients with neurological infections, bloodstream infections, and periprosthetic joint infections (PJIs) (Grumaz et al., 2019; Tarabichi et al., 2018). Additionally, an added value of NGS over 16S PCR followed by Sanger sequencing is the additional potential of information for the prediction of antimicrobial resistance and surveillance data (Gu et al., 2019; Mahmoud et al., 2025). NGS platforms may enable transcriptomic analysis through RNA sequencing, including messenger RNA, allowing the assessment of the expression of antimicrobial resistance genes. However, the presence of a resistance gene does not always correlate with phenotypic resistance due to variable gene expression, regulatory mechanisms, or the presence of non-functional gene variants. In addition, detecting antimicrobial resistance detection by NGS is not yet widely available in routine clinical practice (Hilt and Ferrieri, 2022).

NGS strategies can employ a DNA-centric approach, an RNA-centric approach, or a combination of both approaches. A DNA-centric approach identifies all organisms except RNA viruses. Including an RNA-centric approach further enables the identification of transcriptionally active microorganisms (Simner et al., 2018). However, because RNA is more vulnerable to degradation by RNase that could be present in the specimen, it is important to consider if RNA should be co-purified with DNA. The use of chaotropic agents is effective for removing nucleases, including RNases which protect RNA from degradation (Thatcher, 2015).

With the rapid decline in the cost of sequencing over the last few years, NGS is steadily finding a place in clinical practice and is already beginning to play a critical role in detecting infective organisms (Chiu and Miller, 2019).

Two recent meta-analyses have examined the diagnostic value of NGS in NVO (Higgins et al., 2022). In one of the meta-analyses, five studies including 266 patients were evaluated (Mahmoud et al., 2025). The pooled results showed that NGS had a sensitivity of 82 % (95 % CI 63 %–93 %) and a specificity of 71 % (95 % CI 37 %–91 %) (Mahmoud et al., 2025; Li et al., 2025a). The diagnostic odds ratio was 11 (95 % CI 4–35), and the area under the receiver operating characteristic curve was 0.89 (95 % CI 0.86–0.92; p<0.001 ), indicating moderate to high diagnostic accuracy. In another recent meta-analysis of 770 patients, NGS showed a pooled sensitivity of 81 % and specificity of 75 %, outperforming tissue culture, which had a sensitivity of 34 % and a specificity of 93 % (Li et al., 2025a). In a multi-center study of 301 patients with NVO, NGS achieved a pathogen identification rate of 77.9 % compared with 27.2 % for culture, significantly increasing diagnostic yield. Notably, 175 of the 301 patients (58.1 %) had received prior antibiotics, which likely contributed to the low culture positivity (Li et al., 2025b). The concordance between genotypic resistance determinants and phenotypic antimicrobial susceptibility is inherently variable and is influenced by sequencing depth as well as the underlying biological mechanism of resistance. Many clinically important pathways such as efflux pump overexpression, porin alterations, and chromosomal or regulatory mutations do not correspond to discrete resistance genes and therefore would not be captured by ARG-based detection alone. As illustrated by recent work on *Stenotrophomonas maltophilia*, bacteria frequently rely on intrinsic and multi-factorial resistance mechanisms, including efflux-mediated fluoroquinolone resistance and inducible β -lactamase expression, which emphasize the limits of purely gene-centric approaches. Consequently, resistance predictions generated by NGS should be regarded as hypothesis generating rather than definitive and must be interpreted in conjunction with phenotypic susceptibility testing, pharmacologic considerations, the adequacy of source control, and the patient's clinical response (Rhoads, 2021; Wang et al., 2018). NGS-based resistance profiling should complement, rather than replace, phenotypic antimicrobial susceptibility testing, which remains the standard for guiding antimicrobial therapy.

Another application of NGS is the detection of plasma microbial cell-free DNA (mcfDNA) as implemented in the Karius test, which is particularly advantageous for diagnosing deep-seated infections like NVO. mcfDNA testing can increase the microbiologic yield in osteoarticular infections (OAIs), including NVO, and especially when invasive sampling is unsafe (Petri et al., 2024) because the Karius test only requires a blood specimen. mcfDNA is increasingly being explored in fields such as pediatric infectious diseases and oncology, especially for detecting opportunistic infections in immunocompromised patients (Hogan et al., 2020; Rossoff et al., 2019). However, this assay does not allow for the localization of the infection site and may occasionally detect circulating microbial DNA not associated with active invasive infection rather than true infection. The turnaround time is typically about 1 d from plasma separation, and proper pre-analytic handling including temperature control and timely processing is essential for reliable results. The Karius test is a CLIA-validated laboratory-developed test but is not FDA cleared.

In recent years, several studies have evaluated the use of mcfDNA sequencing for diagnosing OAIs. In a prospective study of 53 patients with PJI, Karius plasma mcfDNA sequencing accurately identified the same pathogens as standard intraoperative joint cultures in 35 cases (66 %), including eight cases in which cultures only provided genus-level identification. Using surgical tissue and synovial fluid cultures as the reference standard, the addition of mcfDNA testing increased the overall diagnostic yield from 87 % to 94 % (Echeverria et al., 2021). In a 2024 retrospective cohort of 73 patients, the Karius test was positive in 22 of 43 (51.2 %) infected cases (Petri et al., 2024). Of these, 11 (50 %) were concordant with conventional diagnostics, and in 5 cases (22.7 %), the Karius test identified unexpected bacterial pathogens. NVO and OAIs with concurrent endocarditis or endovascular infection were significantly associated with higher diagnostic certainty when Karius was used ( P<0.001 and P=0.005 , respectively). The test improved pathogen detection by 11.6 % and aided in achieving a diagnosis, particularly in culture-negative or antibiotic-pretreated NVO (Petri et al., 2024). However, its high cost and limited accessibility currently restrict widespread clinical use, and its role in routine diagnostics remains to be clearly defined.

While several studies have highlighted the use of NGS to complement cultures in the detection of pathogens in patients with suspected NVO, there are some drawbacks to overcome before widespread utilization can be achieved. With NGS, most nucleic acids in the specimen will be human-host-derived, making pathogen detection difficult since the host nucleic acids interfere with microorganism identification. The technique is also susceptible to bacterial, fungal, and exogenous DNA, including laboratory, environmental, and reagent-derived DNA at multiple steps during processing. Interpreting results is challenging as many contaminating organisms are also considered potential pathogens in NVO. Despite its high sensitivity, this technique has limited specificity, as shown by the number of false-positive results due to the DNA amplification of contaminants and the persistence of bacterial DNA after bacterial death. Furthermore, with high sensitivity of these techniques, there is a need to better understand the clinical significance of unusual organisms and their role in infections. In these cases, results should be interpreted within the broader clinical context, integrating clinical presentation, laboratory findings, imaging, and histopathology when available.

Widespread use of this technique is currently limited by its high cost and the complex laboratory and bioinformatic workflows it requires. Interpretation of NGS reads requires bioinformatic pipelines which are also limited by the completeness of reference databases. NGS is generally labor intensive and involves multiple steps, including nucleic acid extraction, library preparation, sequencing, and data analysis. The turnaround time for NGS was reported in multiple studies and ranges from 6 h to 7 d, with an average of 48 h from specimen receipt (Afshinnekoo et al., 2017). This variation depends on the laboratory testing approaches, sequencing technology, methods, and bioinformatics programs.

Optimal patient selection, testing strategies, and the interpretation of NGS results remain incompletely defined, and further studies are needed to establish the appropriate, cost-effective, and clinically meaningful application of NGS (Higgins et al., 2022). While NGS testing is relatively expensive compared to traditional microbiological tests, its cost-effectiveness is context dependent and should be considered alongside clinical utility. In many cases, empiric antimicrobial therapy is effective, and the incremental benefit of pathogen identification may be limited, particularly in clinically stable patients. In 452 orthopedic infection episodes, McBride et al. (2026) found that 16S rRNA sequencing detected bacteria in only 9.5 % of tests and had a limited impact on antimicrobial de-escalation and stewardship, although it was occasionally useful in culture-negative or antibiotic-pretreated cases.

Accordingly, the use of NGS may be most appropriate in selected clinical scenarios, such as patients with severe or progressive disease, immunocompromised hosts, prior antimicrobial exposure, or suspicion of atypical or resistant pathogens. In these settings, pathogen identification may facilitate targeted therapy, antimicrobial de-escalation, and improved stewardship. Conversely, in patients with typical presentations and low risk for resistant or unusual pathogens, a more conservative approach with empiric therapy may be reasonable.

Based on current literature, employing NGS may be justified when conventional methods, which are still considered the gold standard, fail to provide insights into the disease process, thus positioning NGS as a potential last resort for identifying an infectious process after results of other conventional testing have been provided. Alternatively, NGS may be warranted for critically ill or severely immunocompromised patients for whom a prompt diagnosis is essential for better outcomes. Currently, the appropriateness of NGS in routine clinical care is still being actively investigated, and further evidence is needed to establish its use. Table 1 summarizes the strengths and limitations of the diagnostic modalities discussed in this review.

**Table 1:** Strengths and limitations of diagnostic modalities for NVO. Abbreviations: NVO: native vertebral osteomyelitis; IDSA: Infectious Diseases Society of America; ESR: erythrocyte sedimentation rate; CRP: C-reactive protein; NGS: next-generation sequencing; mcfNGS: microbial cell-free DNA next-generation sequencing.

Diagnostic method	Strengths	Limitations
Blood cultures	Non-invasive; recommended first line by IDSA; high specificity when positive	Lower sensitivity than sequencing assays; prior antibiotics affect the results
Image-guided biopsy	Enables direct microbiologic and histopathologic sampling	Invasive; low sensitivity (48 %–52 %)
Open surgical biopsy	Allows direct visualization of tissues and surgical debridement; higher sensitivity than image-guided biopsy	Invasive
Inflammatory biomarkers (ESR, CRP)	High sensitivity; can be used to rule out NVO in the setting of back pain	Poor specificity; limited diagnostic value alone
16S rRNA broad-range PCR	High sensitivity compared to culture; detects fastidious and slow-growing bacteria; less affected by antibiotic pretreatment	Risk of false positives from contamination or non-viable DNA; no antimicrobial susceptibility results; limited in polymicrobial infections
NGS	Unbiased detection of all pathogens including mixed and rare species; higher sensitivity than culture; valuable in culture-negative or antibiotic-pretreated cases; may have the capability of predicting antimicrobial resistance genes in the future	Lower specificity compared to culture; risk of contamination and over-interpretation; higher cost compared to culture
mcfDNA	Non-invasive (blood/plasma specimen only); can aid in diagnosis of culture-negative or antibiotic-pretreated NVO cases	Does not localize infection; high cost; limited accessibility

Future studies should focus on standardizing molecular diagnostic techniques and defining their role in the routine diagnosis of NVO. Multi-center prospective studies comparing 16S rRNA PCR, NGS, and mcfDNA testing with conventional methods are needed to assess the accuracy, cost-effectiveness, and impact of these techniques on clinical outcomes. Further research should also aim to optimize specimen processing, reduce contamination risk, and improve the predictability of molecular results through better bioinformatic tools and reference databases. Such research will also support the ability of these techniques to accurately detect antimicrobial resistance determinants for improved therapeutic guidance.

## 3 Conclusion

NVO remains a diagnostic challenge that requires the combined application of clinical, radiologic, and microbiological information. Culture-based methods continue to be the main diagnostic approach, but molecular techniques such as 16S rRNA PCR, NGS, and mcfDNA testing have improved pathogen detection, especially in culture-negative or antibiotic-treated cases. The early use of NGS-based methods may shorten the time to diagnosis and reduce the need for repeat invasive procedures, although their clinical benefit and cost-effectiveness still require confirmation in larger studies. At present, these molecular tools should complement, not replace, conventional diagnostic techniques. A practical approach that combines molecular testing with careful clinical and imaging evaluation offers the best strategy for improving diagnostic accuracy and patient outcomes in NVO.

## Data Availability

No datasets were used in this article.
